# From the Eurasian Steppes to the Roman Circuses: A Review of Early Development of Horse Breeding and Management

**DOI:** 10.3390/ani11071859

**Published:** 2021-06-22

**Authors:** Weronika Klecel, Elżbieta Martyniuk

**Affiliations:** Department of Animal Genetics and Conservation, Institute of Animal Sciences, Warsaw University of Life Sciences, 02-786 Warsaw, Poland; elzbieta_martyniuk@sggw.edu.pl

**Keywords:** horse domestication, ancient breeding, ancient DNA, genetic differentiation, horse husbandry

## Abstract

**Simple Summary:**

Horses were domesticated later than any other major livestock species. Their role in shaping ancient civilizations cannot be overestimated. As a primary means of transportation, an essential asset in warfare, and later one of the key elements of circus entertainment, horses quickly became luxurious goods. Vast amounts of money were invested in the horse industry resulted resulting in the rapid development of horse breeding and husbandry. This review examines paleogenetic, archeological, and classical studies on managing horses in antiquity. Many ancient approaches and practices in horse management are still relevant today and some of them, now abandoned, are worth re-examination.

**Abstract:**

The domestication of the horse began about 5500 years ago in the Eurasian steppes. In the following millennia horses spread across the ancient world, and their role in transportation and warfare affected every ancient culture. Ownership of horses became an indicator of wealth and social status. The importance of horses led to a growing interest in their breeding and management. Many phenotypic traits, such as height, behavior, and speed potential, have been proven to be a subject of selection; however, the details of ancient breeding practices remain mostly unknown. From the fourth millennium BP, through the Iron Age, many literature sources thoroughly describe horse training systems, as well as various aspects of husbandry, many of which are still in use today. The striking resemblance of ancient and modern equine practices leaves us wondering how much was accomplished through four thousand years of horse breeding.

## 1. Introduction

Over more than 5500 years of domestication [1,2,3], horses performed various roles in human civilization. Before gradual replacement with cars in the 20th century, the horse was a primary means of transportation, as well as a plowing force and a source of various farming products, including milk, meat, and leather [4]. The foundation of horse husbandry and breeding had already been established in antiquity. While the human–horse relationship changed through the centuries, depending on human uses of horses [5], it can be said that due to their special status and multiple values, horses received more attention and affection than any other livestock species [6].

This paper presents a short overview of horse domestication and the history of differentiation of phenotypic traits. Most of all, it aims to describe ancient breeding methods and practices, as well as horse husbandry in antiquity, which has currently received more attention from zooarcheologists than animal scientists.

The primary written sources of information regarding the basic philosophy of breeding and rearing horses in antiquity are the agricultural treaties of various Greek and Latin authors [7,8,9,10,11]. Reviewing historical literature sources with recent DNA sequence discoveries helps us to understand how many changes in horse phenotype over the years were intentional and how many resulted from natural selection, random genetic drift, or other non-human-conducted processes.

Finally, the remarks of ancient authors on various aspects of horse husbandry have been evaluated. This includes breeding techniques, herd management, foal raising, feeding, training stages, and veterinary medicine. Ancient practices are compared to modern ones, leading to an impression of surprising similarity.

## 2. Horse Domestication and the Beginning of Horse Riding

The first evidence of the interaction between humans and horses dates to the Lower Paleolithic period and comes from Schöningen, Germany, where numerous smashed and butchered carcasses, as well as horse bone tools, were found [12]. Initially, horses were hunted for meat and hides [13]. As an example of intensive hunting use of horses, in a small ancient hunting area in Solutré, France, the bones of between ten thousand [14] and one hundred thousand [15] horses were found.

Despite the apparent intensive trophic interactions between humans and horses in the Pleistocene, there is no domestication evidence before 6000 years ago, making horses the last of five most common livestock species to be domesticated [1]. The debate remains with some believing domestication took place in single culture and then spread across the world, while others support the view it was developed in numerous cultures independently [3]. Recent paleogenomic studies on mitochondrial diversity support the latter hypothesis [16,17], claiming that the domestication process was likely taking place in the Eurasian steps within at least two different cultures.

There is both genomic [18] and archeological [19,20,21] evidence proving horse domestication in the Botai culture in Northern Kazakhstan (5600–5000 BP) [2], such as corral enclosures and manure management, mare’s milk residue in ceramics, morphological changes in metacarpal bones, and others. Although the evidence for the Botai horse domestication is strong, horses from this culture contributed to the genetic makeup of the modern domestic horses in a very limited way (between 2.0% and 3.8%) [16].

Archeological discoveries from Dereivka (Dnieper region, 5950–5530 BP) [17], reveal the presence of complete horse skeletons; however, some of them were carbon-dated to a much younger period (2790 BP–70 AD) [22]. The absence of old animals and an overwhelming majority of males [23,24], may suggest that horses there were rather raised than hunted for meat [25], but could also point to the specific hunting strategies [17].

**Figure 1:**
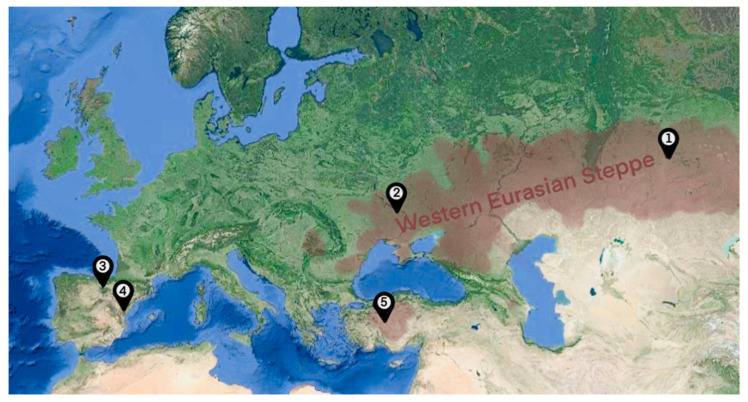
Locations of the putative centers of horse domestication: 1—Botai [21], 2—Dereivka [24], 3 and 4—Iberia [26], 5—Anatolia [29], and the approximate reach of Western Eurasian Steppe in the Bronze Age.

Other studies proposed geographically distant putative independent domestication centers, such as Iberia [26] and Anatolia [27]. The newest research, based on extensive genomic studies [28,29], has shown that horses from these regions contributed limited ancestry to modern horses (Figure 1).

Wheeled means of transportation began to appear in the Eurasian steppes between 5600 and 4300 BP [30,31]. It is widely accepted that first draft animals belonged to the bovine family and there is no evidence of draft horses being used at that time [31]. The first undeniable evidence of horse-powered transportation—light, two-wheeled chariots—comes from the Sintashta–Petrovka archeological site, and dates to c. 4100–3700 BP [30,31,32,33,34]. The chariot graves, where, together with humans, horses and chariots were buried as a sacrificial deposit, indicate importance of horses in sacral aspect of the culture. By the end of the fourth millennium BP, harnessed warriors were widely spread across most ancient civilizations, leading to the conclusion that chariots must have gained a substantial popularity.

The vital and still not fully answered questions are why, how, when, and where a human has mounted a horse for the first time. Older research supported the hypothesis that this event took place at the very beginning of domestication. Anthony et al. [35] tried to determine the origin of horse riding based on mesial bevel of the premolars, which could be a sign of bit-wear. However, this method was questioned by other researchers [36] and cannot be considered a reliable tool. At present, there are widely held views that horse riding spread more recently (by the end of the Bronze Age) [30].

From the late fifth millennium BP, both humans and horses were painted, carved, and sculptured, but none of the many finds represent a man sitting on a horse. The earliest representation of mounted equids dates to c. 4500 BP in Mesopotamia [37]. However, the poor state of preservation prevents the determination of the exact equine species. The first unquestionable iconographical proof of horse-riding was found in 1991 on a royal seal belonging to Abbakalla of Ur (the scribe in the service of King Shu-Shin), which dates to the beginning of the fourth millennium BP [37,38]. Many other early horse-riding iconography pieces show the rather uncomfortable riding style while sitting on the animal’s rear. Some researchers consider this fact as proof that the idea of mounting a horse was inspired by donkey-riding, which could have been developed earlier [37,39]. Rossel et al. [40] state that using the donkey as a mean of transportation was common in the First Dynasty Abydos (c. 5000 BP). It is still unclear why the period between the beginning of the domestication and the development of riding was shorter in the case of donkeys than horses. The possible reason of this phenomenon might simply be explained by the smaller size of the donkeys or their easier tamability, as demonstrated by the study on Afar communities in Ethiopia [41].

## 3. Spreading of Horses in the Ancient World

As mentioned before, from the sixth millennium BP oxen’s usage as draught animals was common in the Near East. The first vehicles were heavy and usually equipped with four disc wheels, and both their speed and range were very limited [42]. Subsequently, the oxen were replaced with equids—firstly donkeys or ass-onager hybrids [35], then horses. The invention of spoked-wheel light chariots c. 4000–3800 BP was one of the ancient era’s milestones, as it increased the speed of traveling through steppes from two to ten miles per hour [43] and laid the foundation for their further military use [44].

In the fifth millennium BP, the use of horses in transportation rapidly spread within the known world, as they most likely accompanied tribes migrating from the steppes [45]. Horses were also the subject of trade; in about 4100–4000 BP, they probably reached and spread across Central Asia, a region where only onagers appeared in feral herds [46]. A new exotic animal quickly became a subject of interest for the aristocracy, and was represented in various pieces of art [47]. The horse-drawn chariots appeared in the Near East about 3800 BP, as represented on Syrian seals [48]. Other discoveries confirmed the horse presence in the area of Southern Iran at that time [49].

The possession of the fast and swift chariots gave the peoples of steppes a tremendous military advantage [47]. Increasing welfare of horse owners led to the birth of the horse industry: 3800-year old clay tablets found in northeast Syria mention the teams of horsemen, trainers, and grooms. Another text from this period describes the import of valuable specimens from Syria and Anatolia to Mari and Assyria [50].

The osteological evidence suggests that horses were a significant factor in Troy’s civilization, which perhaps served as a horse market as well as a training and breeding center [51]. The reading of Homer’s *Iliade* indicates the crucial role of horses in the Trojan War that cannot be denied.

One can also find the believed oldest description of horse racing as an entertaining event in Homer’s epic story. Chariot racing (synōris) was a part of Patroklos’ funeral games and, as described by the poet, the noblest one. In the story, Antilochos manages to win over Menelaos despite having slower horses using a strategy advised by his father, Nestor. Therefore, it can be concluded that Homer considers the environmental factor of the driver, the significance of which has been confirmed by various modern studies investigating the jockey’s effect on racing performance of horses [52,53,54].

**Figure 2:**
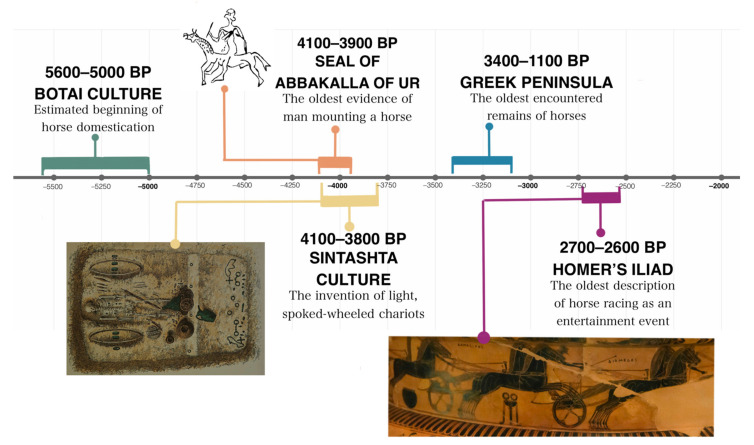
Milestones in horse industry development BCE.

It was not a long distance for horses to reach mainland Greece from the neighboring Troy. However, Donaghy [1] suggests that it was probably not the only path for horse migration, another one being from the North, via Macedon and Thrace. The first osteological evidence of horses’ presence in the Greek peninsula dates to 3400–3100 BP [55]. By the end of the ancient era, horses were present across the entire known world (Figure 2).

## 4. The Influence of Warfare on Breeding Criteria

Anthony and Brown [56] stated that the horseback inter-tribal incursions, which ultimately shaped the socio-political situation of Eurasia [57], began as early as domestication. This was thousands of years before the development of the famous Iron Age cavalry. However, there is no evidence of specialized Eneolithic weapons for mounted warriors, which suggests that assaults on horseback might have been relatively ineffective. There is a hypothesis that, in tribal raiding strategies, horses were used just to escape after attacks, which corresponds with the tactic used by Native Americans [58]. If that was the primary role of horses, then raiding horses were likely chosen for exceptional speed on short distances.

The invention of the chariot changed the dynamics of tribal wars in the steppes. It provided an elevated platform from which javelins were thrown. It also intimidated the enemies by their speed and loudness [47]. Given the advantages of chariots, it is not surprising that their use in warfare was quickly adapted by most ancient cultures.

Contrary to other Iron Age civilizations, Greece, famous for its infantry, and later for its cavalry, had not employed chariots during the battle [59,60,61,62]. Nevertheless, chariots were an essential means of transportation both for soldiers and messengers, and there is some evidence that they also served for certain military purposes. Some researchers find an apparent parallel between the use of chariots in Greece and later in Britain during Caesarian times [59], an interesting fact in the context of these nations’ role in development of horse racing. The chariot fighting style in Greece and Britain required speed and overall superior swiftness of the horses, which likely would have been an important factor in selective breeding.

With the exclusion of the people of the Eurasian steppes, such as Scythians, horse riding seems to be known but not widely spread in the Mycenaean times [63]. There are some descriptions of Trojan War heroes mounting a horse in Homer’s *Iliade*, but how common the mounted warriors were in these times is yet to be determined [64]. Cavalry forces fighting from horseback were not popular in Greek art until 2500 BP. Accordingly, it can be assumed that until then, chariots wer much more widely used than the cavalry [65].

## 5. Breeding Objectives

The typical feral phenotype traits of horses are stockiness, relatively small body, erect mane, dun coat color and primitive markings (dorsal stripe, horizontal legs striping and lighter edges of dark mane and tail). These traits are presently observed in various primitive, semi-feral breeds of the domestic horse [66,67,68]. They are also typical for Przewalski’s horses (phylogenetically separated from modern domestic horses [69,70] and proven to be descendants of Botai-like individuals [16]), as well as for the other feral species of *Equus* family, such as zebras, wild donkeys, or onagers.

Archeological and literature studies confirm the existence of light-boned, flowing-maned horse populations of various colors in Central Asia in the fourth millennium BP [71,72]. There is also evidence of horses phenotypically similar to modern Arabian horses in Egypt between 4000 and 3300 BP [73,74]. These horses were often described as “superior”. The new mutations in phenotypic feral traits were most likely favored by breeders since the early stage of domestication and they might have been a subject of directional selection.

**Figure 3:**
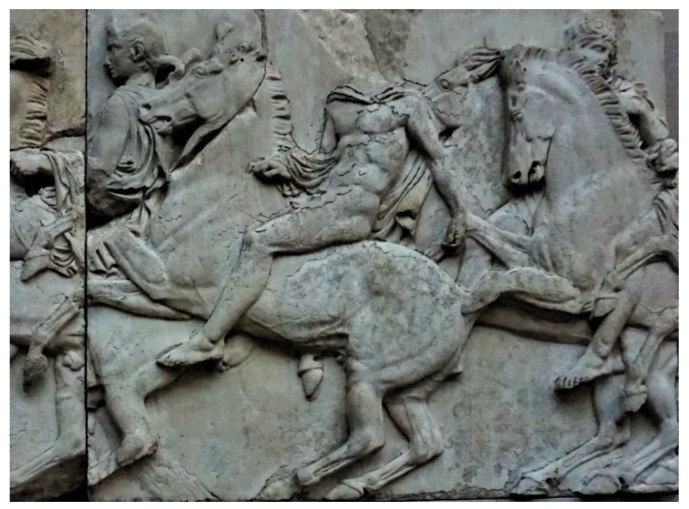
Horses of the Partenon Friese. Note that the horses are ridden bridle less and bareback. Wikipedia Commons.

The popularity of horses in the Bronze Age has been reflected in many art pieces. Despite numerous genomic studies, iconography remains an important source of information about the conformation of ancient individuals. The most famous portrait of Greek horses is found in the Parthenon Frieze (2438–2432 years ago). It captures the general conformation type: a large head with a flat profile, short, thick neck (probable remains of the primitive ancestors), a short stocky body, and relatively long legs with remarkably well-developed tendons and joints (Figure 3).

Although there is a little primary source of information about selection criteria in ancient Greece, the phenotype traits visible on the frieze compared to the primitive horse’s phenotype allow us to make some assumptions about breeding directions at the time. As the horse value was based on its usefulness in battle, the important qualities would have likely included excellent health and endurance, in addition to speed and agility. However, horses with noted battle merits are usually described by the ancient authors only in vague superlatives as “fine” or “excellent,” without detailed information about their conformation traits [7,8,9].

### 5.1. Overall Conformation

In De Re Rustica, Varro describes in detail the ideal conformation of the breeding stock. Mares should have “broad quarters and bellies”, while stallions “broad body, handsomeness, with no part of the body breaking the harmony” [9]. While the correct pelvis anatomy of the mare is thought to be essential, although not scientifically proven, for a seamless delivery of foals, the conformation criteria for stallions indicate Varro’s more profound breeding thoughts. Naturally, conformation correctness is necessary for selecting a stallion for breeding, but even today many chief sires of various breeds possess certain faults that depart far from harmony and handsomeness [75,76].

The parts of conformation that underwent the most significant changes are the limbs. The measurements of Scythian horses’ metacarpals revealed significantly higher slenderness compared to modern primitive horses [77]. The selection for this trait was later confirmed by a genomic study, which showed an overrepresentation of genes having expression in the anteroposterior axis, carpal bones, the tibia, the thoracic sling, and the radius bone [78].

### 5.2. Height at Withers

During the first two millennia of domestication, the horses of the Eurasian steppe were relatively small, measuring between 122 and 152 cm (12.0–15.0 hands) in withers (with the average of 137) [33,58]. For a long period of time, these small horses were in the majority across Europe and the Near East [46]. The small size limited, or even precluded, their ability to be ridden. As stated by Herodotus, the ponies of Sigynnae were incapable of bearing riders, but very swift in pulling chariots. However, according to the archeological evidence, most of these horses were Botai-like, and hence, the ancestors of today’s Przewalski’s horses.

Given the estimated heritability of the height in withers trait ~0.72 [79], it can be assumed that one of the most important breeding goals in the ancient era was to enlarge the overall height of horses. However, Varro stated that horses used for breeding should be neither over nor under size (quod nec vastos nec minutos decet esse) [9,80]. The average Roman chariot horse measured between 135 and 155 cm in withers [81]. Therefore, increasing the height in withers must not have been the sole breeding goal in the antiquity.

As in other domesticated species such as dogs and cattle, in horses, height in withers is determined by a few loci, contrary to the height in humans. To this date, two quantitive trait loci (QTL) responsible for height have been identified in horses, with two more loci having a smaller effect [79]. The frequency of three out of five mutations: chr9:74795013, chr9: 74795236, and chr9:74798143 in the *ZFAT* locus rapidly grew in the first millennia of domestication, reaching their peak frequency of ~0.6–0.75 around 3500 BP [28]. Interestingly, most of these alleles seem to be the subject of a negative selection in modern breeding.

### 5.3. Coat Color

Domestic animals usually show significantly higher variation in colors and patterns than wild species. Variation in a horse’s coat color seems to be one of the first traits of interest in early human selection [82], as the rare color phenotypes were associated with a higher prestige [83].

Currently, 23 genes are identified as responsible for inheritance of equine coat colors. Basic colors: bay, chestnut, and black, are controlled by the interaction between two genes: Melanocortin 1 Receptor (*MC1R*) and Agouti Signaling Protein (*ASIP*), traditionally called Extension (E) and Agouti (A). To obtain bay phenotype at least one dominant allele in both loci is required. The black phenotype is the result of the interaction between homozygous aa in the Agouti locus and at least one dominant allele in the Extension locus. The chestnut results from the homozygous recessive Extension locus, as shown in Table 1.

Other genes are responsible either for dilutions of the coat color or for the white spotting patterns. Among the dilution genes, only the *Dun* gene in the *TBX3* locus, causing dilution of the basic coat color and often associated with primitive markings, was identified before the domestication. The frequency of the wild-type allele, *Dn^+^* or *Dn^D^*, is the highest in the primitive breeds such as Fjord, and in Przewalski’s horses [84].

Ludwig et al. [85] investigated DNA sequence polymorphisms in fossil horses, analyzing eight mutations in six genes responsible for coat color. There was no variation in Pleistocene horses, suggesting the population was homogeneously wild type bay or bay-dun. The first recessive mutation appeared in the *ASIP* gene in the early Holocene, resulting in the black colored horses. According to Ludwig et al. [85], there is no evidence of human selection at that time. Interestingly, Pruvost et al. [86] found not only bay and black but also leopard phenotypes by genotyping the Western European samples of pre-domestic Pleistocene animals. The leopard spotting is determined by an LP co-dominant allele in the *TRPM1* locus. Other coat-color loci showed no variation in Pleistocene samples. These results confirmed that cave paintings from the Pleistocene era could represent actual phenotypes rather than artistic imagination.

Rapid increases in coat color variation of Siberian and East European horses began around 5000 years ago. The chestnut phenotype (*MC1R* gene in Extension locus) was identified in that period, and it seems to be favored by Copper Age breeders, as the ee genotype frequency reached 0.28 about one thousand years later. Grey horses were identified in Bronze Age samples but were absent in the Iron Age material, which is an interesting case, as the progressive greying with age mutation in locus *STX17* is autosomal dominant and epistatic to all colors [82]. Samples from the Scythian culture (c. 2700 BP) revealed even greater diversity in colors, including cream, black, spotted, bay, and chestnut individuals [78].

Although it is impossible to prove the selection criteria for coat coloration, the continuous increase in the frequency of *ASIP* and *MC1R* mutations, which was observed from the Copper to Iron Age, suggests that the selection towards desired phenotypes took place in this period.

The foundation of the famous farm-fox experiment in Novosibirsk [88] was based on the hypothesis that a critical factor in the domestication process was adaptability for domestication, or tamability, determined by the genetics of behavioral traits. It is most likely that early breeding was aimed at domesticated species to reduce aggressiveness [87] and fear towards humans [83]. Despite being a herbivore with elopement as a primary survival strategy [89,90], horses, especially mature stallions, can also be dangerous and aggressive.

Positive selection for behavior in the early stages of domestication seems unquestionable, as the genome analysis revealed significant enrichment in genes involved in abnormal synaptic transmissions, associative learning, and reward, but also for oxytocin secretion [78]. Interestingly, many studies on different species have revealed a correlation between tamability and increased variation of coat colors [90,91,92] as well as between certain behavior traits and allele distribution in coat color *loci*. In horses, the association between self-reliance and the genotype in the *ASIP* locus has been identified [93,94]. The precise molecular mechanisms linking behavior and color phenotypes, have yet to be found.

### 5.4. Racing Performance

As previously stated, horse racing competitions, either driven or mounted, were popular in the Iron Age. Mutations in genes associated with horse racing performance, such as *ACTN*9, *CKM*, *COX4/1*, and *COX4/2*, were reported even in samples of mid-Holocene and Upper Paleolithic horses [95,96]. However, the most significant mutation in the *MSTN* gene, responsible for muscle hypertrophy and speed performance, was only found in Bronze and Iron Age samples [78], and its frequency rose significantly only in the last millennium [28]. This discovery may indicate that around 4100 BP, breeders began to select horses not only for their endurance but also for high-speed potential, still the intensification of this breeding direction is relatively new and cannot be associated with antiquity.

The key question follows, if racing potential was evaluated on the breeding stock own performance, as it is in many cases today, or if it was also tested on their progeny. Varro’s comment that “some horses are fitted (…) for breeding, and others for racing”, and most importantly, the fact that the best individuals raced until 20 years of age [97] can lead us to two opposite conclusions:

- Ancient horsemen were unaware of heredity, and so-called breeding was in fact, just reproduction to obtain animals for further use.
- Racehorses’ evaluation for breeding was based solely on progeny performance. Most valuable individuals of both sexes were kept for breeding and not raced to prevent injuries at the racetrack.

Moreover, sources demonstrate that Roman charioteers favored horses imported from Africa (Libya) [98]. The genetic specification of this particular subpopulation of favored horses is yet to be determined.

## 6. Breeding Practices

### 6.1. Inbreeding

A genome study of Berel horses (third millennium BP) revealed a surprisingly low inbreeding level [78]. This may or may not put into question the practice of intentional, human-conducted mating in ancient times. Monard and Duncan [99] demonstrated that feral horses possess a natural mechanism to avoid inbreeding: between 1 and 3 years of age, fillies are forced to migrate to another herd with familiar females but unfamiliar stallions, while colts gather in bachelor bands of multiple origin. These two discoveries still cannot answer the question as to whether Berel breeders intentionally avoided breeding related animals or did not interfere with the natural herd structure and reproductive behavior. The most plausible explanation is that the mating occurred in a much more flexible way, and, as a result, the reproductive success of every individual was more uniform than it is today. More information about inbreeding comes from the Iron Age. Aristotle and Latin authors repeatedly told stories of stallions being ashamed of mounting their dams or daughters [10]. While such stories are of course fictional, they suggest that ancient horsemen held negative views on incestuous mating.

### 6.2. Genetic Diversity

During the first millennia of domestication, horses showed a high level of genetic diversity. Studies on nuclear DNA demonstrated significant gene flow between domestic and wild populations [100,101]. Some studies on ancient mtDNA have demonstrated that, in the creating of a domestic population, between 17 and 46 individual maternal lineages were used [102,103]. The genetic diversity of the Y chromosome raises a few more questions. Initially, it was believed that only a few paternal lines contributed to the forming of the modern domestic stock [104,105]; however, recent discoveries demonstrated that the loss of Y genetic diversity could have occurred more recently [106,107].

A study by Fages et al. [28] demonstrated a ~16.4% median drop in individual heterozygosity levels in modern breeds compared to horses that lived more than 200 years ago. A massive loss of genetic diversity resulted in the accumulation of deleterious variants. Key questions arise: what levels of population size were considered sufficient by ancient breeders, and how significant was the impact of natural negative selection of deleterious mutations?

The reduction of paternal lines diversity might result in some positive outcomes. For instance, a susceptibility to EAV infection, determined by the *CXCL16* gene, was significantly reduced in the last 200 years [28].

### 6.3. Hybridization

A genomic study by Fages et al. [28] identified two mules dating from the Iron Age. Mules, hybrids between ass’ jacks, and horse mares, are known for their strength, resistance, and low maintenance. However, they are sterile and, therefore, used only for work, especially as beasts of burden. Mules are also portrayed in Egyptian iconography, usually as ploughing animals, and frequently collated with “Pharaoh’s horses” to underline the differences in conformation between mule and horse [73]. Varro makes an interesting statement claiming several cases known in the Roman Empire of mule mares bearing a foal [9]. The opposite direction of crossing, i.e., a stallion with an ass’ jenny, was also known in Roman times.

## 7. Stud Management and Horse Husbandry in Classical Antiquity

After the development of the system of capturing and taming feral horses, the next step in domestication aimed to find the optimal way to manage the captured herds. This includes: determination of the right size of the herd, the sex ratio, reproduction strategies, and later various elements of husbandry, such as feeding and raising the young stock. As the evidence for certain practices can only be found in written or iconographical sources, this part of the paper focuses on the classical period of antiquity and mainly on Greek and Latin cultures.

### 7.1. Herd Size and Management

As previously stated, during the early phase of domestication, horses were kept in corrals and most likely bred within family groups. There is no information available on herd sizes from the first Eurasian steppe horse keepers, but according to modern Mongolian and Northern Kazakhstan tribes, 10 horses are sufficient to maintain one family [108]. Taking under the consideration the stallion’s physiological abilities to breed, it is safe to assume that one family group contained, as it is today, one stallion, and 10–15 mares with their offspring. The surplus progeny could either be slaughtered for meat or moved to another family group.

The size of the herd and the breeding practices were strongly dependent on the type of horses being produced. Columella identifies three breeding directions: elite horses bred for the circus and the Sacred Games; horses used for producing mules; and the common (“ordinary”) stock, which probably served military purposes [8]. This statement is supported by Varro, who points out that choosing and rearing horses for the army and for racing shall be based on different criteria [9].

Herd size was also determined by the owner’s welfare and, as it is today, the forage base. Varro warns the breeders that feeding too many horses on a given area of pastures can lead to losing both animals and profit [9].

Recent study showed no special preference by early horse herders for horses of a given sex until c. 3900 BP, when males began to appear much more often in osteological residues in burial sides, suggesting male-oriented preferences of Iron Age breeders [13]. Moreover, another study revealed much larger Y chromosome diversity, especially in Roman and Gallo-Roman horses [28], than in the modern population. This may indicate a different approach to selecting stallions than mares and even equal reproductive success of both sexes.

### 7.2. Stock’s Herd Life

The minimal age for mating given by ancient authors is generally consistent with modern knowledge: both Pliny and Aristotle state that mares and stallions are capable of mating at two years of age; however, the offspring tend to be small and weak, so it is recommended not to mate the horses until they reach the age of three [7]. Modern breeding practices still reflect this approach.

The opinions on the upper breeding age limit are less consistent and sometimes confusing. Aristotle claims that a mare can successfully produce a foal until the age of forty, while a stallion can be used at the stud up to 33 years of age [7]. On the contrary, the Latin authors argue that a mare is suitable for breeding until ten years of age [10]. Both views are hard to accept in modern breeding, as horses rarely reach the age of forty, and the age of 10 is considered the optimal rather than rearmost age for a mare to be bred [109]. The exaggerated periods of breeding life can also be caused by inaccurate criteria for age determination. There is no information about the existence of any form of breeding records in antiquity, and the age estimation based on teeth in antiquity was based upon false assumptions [10]. However, for the sires, the age limit put by the ancient authors is consistent with modern practices, i.e., stallions can breed as long as they have the interest in mounting the mare [11].

### 7.3. Breeding Season

The horse is a seasonal polyestrous species. The ovarian activity in mares is affected by the period of day light, which varies with longitude. In the Northern Hemisphere, the natural breeding season lasts from April to September [110]. In artificial environments, for economic reasons, breeding can begin in February and end at the end of July. Varro recommends beginning mating at the vernal equinox (20 March) and continuing until the (summer) solstice (21 June) [9]. Both approaches are based on the same intention: a foal is born at a convenient time and can fully profit from the pasture season and good weather. Columella, on the other hand, claims that for the common horses, “no fixed seasons are observed for breeding” [8].

### 7.4. Covering Methods and Techniques

Two techniques used in antiquity can be described as harem mating and assisted live covering. The first was used to produce “common horses” for everyday work [8]. Matings of more valuable animals were performed by bringing the stallion twice a day (in the morning and the evening) to a tied mare, with a groom supervising the intercourse [9].

During covering, the major problem occurs when one of the parties is not interested in mating. In this case, the mare usually defends herself by kicking. Apart from tying the mare’s legs [9], a teaser stallion can be introduced beforehand to arouse the female [8]. Both methods are still used today. Varro proposes an interesting solution for arousing the stallion: that is to mix the center of the squill bulb with water and mare’s vaginal discharge and spread the mixture over the stallion’s nostrils [9]. Squill (*Drimia maritima* L.) is a perennial bulbous plant species used in traditional medicine since c. 3500 BP [111]. None of its active compounds, including scillarenin, flavonoids, phytosterols, and bufadienolides, possess a proven impact on reproductive performance; therefore, this ancient practice is worthy of further investigation.

### 7.5. Foal Raising and Weaning

In Roman times, foals were led out to the pasture ten days after birth and remained there for the first five months of their lives [9]. After that stage, they were led back to the stable, where they stayed with their dam until they reached a minimum two years of age.

Both Aristotle and the Latin authors agreed that it is essential to maintain at least a two-year gap between foalings, to allow a foal to fully benefit from the dam’s milk. This is an approach that is difficult to understand. In general, the lactation period in mares lasts about 180 days. After that time, the role of milk in a foal’s nutrition becomes marginal. Today, six month old foals are usually weaned from the dam, while feral foals naturally separate from their mother by the age of 8–9 months [112]. The ancient’s view on the beneficial impact of a two-year gap between foalings is not supported in modern breeding, mostly due to economic reasons.

Moreover, many ancient authors [113] such as Varro, claim that it is practical to wean some foals immediately after birth (hence, euthanize them) for other foals to “grow better”, which might be a solution in the case of a limited forage base.

### 7.6. Feeding

The detailed diet of the Hittite chariot horse can be found in the so-called “Kikkuli text,” Hittite tablets from the fourth millennium BP [114]. The diet included a limited portion of grass and legume plants, cereals such as barley (sometimes boiled), and pasture grazing at specific periods of the day or night. Water access has been limited to once a day or less, although the author apparently understood the importance of electrolytes, providing instructions to give horses salted or malt water after strenuous exercise.

Varro states correctly that pasture grass (grassy plain), which can be exchanged for dry hay in the stalls, is the best forage for breeding stock.

High protein/energy feed most commonly used in the Roman Empire seems to be barley and unidentified “beans”, but ancient breeders do not seem to be particular about that matter. Varro provides the instructions to give the horse “whatever product of the soil they relish” [9]. Ancient breeders also recognized differences in demand between specific groups in the herd, what we call today feeding groups: for example, there was an additional ration of barley for mares with foals or a particular “mixed forage” for youngsters undergoing the first stage of training.

## 8. Development of the Horse Industry in Classical Antiquity

The spreading of horses from the Eurasian steppes into other parts of Europe resulted in their exchange and trade. Over time, horses became a luxury good, and in the Iron Age, they were reserved to the richest part of society. The average cost of a cavalry horse in Greece, a country not particularly geographically suited for raising horses, reached about five hundred drachmae. For that price, one could purchase ten cows or three slaves [64,115]. That amount was also equal to one and a half year’s pay of the average Iron Age Athenian. In the Roman Empire, the average horse price of 125 denarii [116] was equal to a secretary’s seven monthly payments or a private soldier’s six monthly payments [117]. We also know that despite good road infrastructure, most civilian Romans travelled on foot [118]. Given that the only purpose of horse breeding and racing was to provide prestige for the owner through competition-winning, that activity must have been reserved for the highest classes of the society [119]. Due to high value, horse possession required special expertise, which led to the development of many new professions to cover various aspects of horse utilization.

### 8.1. Veterinary Medicine

The exact time and place of the beginning of hippiatrics are unknown. The first documented case of the “horse doctor” is Metrodoros from Lamia in Thessaly c. 2130 BP), but it is safe to assume that this profession existed long before him [120]. Accordingly, there is no exaggeration in saying that the foundation of modern hippiatrics reaches back to the ancient era [121], and that no other species received as much medical attention as the horse.

The source texts on horse medicine can be divided into two categories: the agricultural treaties, where medical practices and treatment are mentioned in a broader context of rearing horses, or even farm management, and the monographies dedicated to veterinary medicine. Works of Latin authors such as Varro [9] and Columella [8] belong to the first category. Similar to many others, they were influenced by the earlier authors, for example Mago from Carthage. As for the medical monographies, the three most important were written in a relatively short period, between 1670 and 1550 BP, and belong to Chiron and Apsyrtos (Mulomedicina Chironi), Pelagonius (Ars Veterinaria), and Vegetius (Digesta Artis Mulomedicinae) [120]. Ancient Greek texts on horse medicine were collected by Oder and Hoppe in one anthology [122,123].

The social status of veterinary practitioners in the later Roman Empire varied immensely. They could be divided into three main groups: slaves, working mainly for the post office and sometimes the circus; free practitioners who had some restrictions regarding their charging for services rendered; and veterinary surgeons, hired usually by the army, and considered prestigious members of the society. The prestige of the role is demonstrated by the fact that the 4th-century medicus veterinarius, Theomnestos, was a friend to the emperor Licinus. On the other hand, Vegetius describes the veterinary job as “a little sordid” [120]. Besides that, early Latin authors, Varro [9], and Columella [8] differentiate between veterinarius, a full-time animal health specialist, and pastor diligens, who possessed some knowledge on curing diseases and could sometimes replace a physician.

The treatment of diseases was based mostly on specific natural medicines and physiotherapy. For instance, one of the most critical diseases typical for horses, laminitis, has been known and treated at least since Greek times [121]. Varro gives us a detailed description of the leading cause and treatment of extertional rhabdomyolisis (ER), or so-called “tying-up”. He correctly states that it occurs when a horse is given feed or water immediately after the exercise, which causes fever and pain. The treatment proposed is to drench an animal with water, rub down with oil and wine, cover it with a blanket, and restrain it from any food. This procedure is almost identical to current treatment.

In the 4th century, two fundamental treaties were written: Pelagonius’ Ars Veterinaria, and Mulomedicina Chironis by an unidentified author known as Publius Vegetius Renatus. Both works were renewed many times over the centuries and provided a strong foundation for modern veterinary medicine.

### 8.2. Training

The oldest evidence of a horse training system dates to the fourth millennium BP. As previously indicated, the tablets from North Syria describe organized teams of charioteers, trainers, and stable staff. The other tablets mentioned early in this paper, the so-called “Kikkuli Text”, found in the territory of modern Turkey, by that time occupied by the Hittite people, described in detail chariot horses training methods [114]. The training program lasted 214 days and, in addition to earlier mentioned feeding instructions, contained a schedule of various activities including interval training, swimming, massages, blanketing, clipping, turning out to paddocks, and other elements, well-known to modern horse trainers. At the beginning of the 1990s, six Arabian horses underwent the Kikkuli training program, and, as stated by the researcher, the resulting endurance improvement was incomparable to any modern programs [124].

Roman horsemen desensitized their horses from an early age to handling and harnesses [9]. At three years of age, the youngsters were broken under the saddle, and light exercise was introduced [9,10,12]. At five years of age, circus horses underwent an evaluation, after which they were placed in one of two chariot positions and began their racing careers, sometimes lasting up to 15 years.

### 8.3. Stable Staff

We know little about horse professionals of pre-Roman times, even regarding ancient Greece. All credit for victories was given to the horse owner rather than trainers [125], even if the owner’s involvement was limited to sponsoring the team [80]. It was during the Roman Empire that chariot racing evolved from being a sign of the owner’s prestige to a robust, prosperous business with hundreds of staff [80]. Racing stables, named factions (factiones in Latin), strongly resembled today’s most prominent international racing operations. Using resources provided by the sponsor (editor ludi), factions bred, trained and took care of racing horses on a daily basis. Each employee had their professional function similar to those known today. Potter [126] lists among them: stablehand (stelarius), cobbler (sutor), groom (conditor), operation manager, and many others specialized in handling horses during races. It is also worth noticing that in Rome, charioteers and horses themselves became popular in the mass culture and commemorated with monuments and epigrams [80].

### 8.4. Competitions

In 2680 BP, the first equestrian event, a four-horse chariot race tethrippon was introduced at the Olympic Games. Mounted racing (kelēs) joined the program 32 years later [127]. Interestingly, jockeys in these races were usually very young boys because of their small body weight. The other equestrian events at the Olympia included kalpē (a race for mares ending with riders running alongside horses in the final lap) and polikon races, apparently reserved for two-year-old horses [97]. Many kinds of races were derived from battle strategies, but it can be assumed that the races reserved for mares or youngsters were part of a breeding evaluation. It is also suspected that some of the Hellenistic countries possessed horses with significantly earlier maturity age, a trait characteristic for modern thoroughbred horses [128].

Between 2648 and 2344 BP, 15 out of 45 winners of tethrippon races were Spartan [2]. Such statistics indicate high levels of training and successful racehorse breeding practices employed in Sparta. Donaghy [1] points out that the Spartan breeding center, a region of Eurotas, was environmentally well-suited for livestock keeping. Due to the small area, rearing practices and the selection criteria must have been strict, which led to fast breeding progress. Spartans also engaged in numerous internal equestrian festivals every year, so the level of training and experience of their horsemen was notable compared to other *poleis*.

Chariot racing gained immense popularity in the Roman Empire. Recent studies underline Etruscan rather than Greek influence in developing chariot racing in the Iron Age [129]. Chariot racing was the oldest and longest-lived of all Roman circus spectacula, having both entertaining and sacred functions in the Roman society [130]. The operation scale of chariot racing can be compared to modern horse racing, as more than sixty days of games were held annually, with 24 races on the card every day [131]. This is an astonishing number of races with more than one thousand horses competing each day, in only one of the dozens of circuses operating within the Empire. As Christianity advanced, the church deemed horse racing as one of the greatest dangers to the new religion [132,133], which eventually led to its gradual loss of popularity and importance.

## 9. Conclusions

The horse was domesticated about 5500 BP in the Eurasian steppes as a source of meat and secondary products, such as milk. With the invention of light, spoked-wheel chariots around 4000 BP, chariot driven horses became a primary means of transportation and an asset in warfare. The importance of horses and chariots and the substantial cost and expertise needed for their management led to the development of specialized horse-related professions.

Horse management from the Early to Middle Bronze eras is not well-documented and leaves many questions regarding horse use and training. The first nomadic horse-keepers most likely managed their herds in a non-invasive way, by maintaining them in family groups and letting them breed within these groups.

The quality of a horse was measured mainly by its usefulness in the battle, and later, especially in the Roman Empire, its racing performance. Various traits associated with warfare and racing were proven to be a subject of selection in the ancient era, including height at the withers, limb development, behavior, and high-speed potential. It is challenging, however, to determine the specific methods of selection used by ancient breeders. From the works of ancient authors, it can be concluded that the breeding value evaluation was based primarily on the conformation of animals and general impression, rather than their utility or pedigree. Moreover, it seems that in the Roman Empire, horse herds used for breeding and horses kept for other purposes including riding or performances in circuses, were kept separately.

Apart from transportation and use in battles, horses were also widely used for racing, which derived directly from war maneuvers. The scale of horse racing in the Iron Age was comparable to today’s racing in Great Britain and France, with its role in society even more significant than today. Furthermore, it is worth noting that the Greeks and Romans were aware of the importance of factors such as rider/driver weight carried by the horse, as well as their skills, the significance of which is still being researched today.

Finally, the relevance of Greek and Roman horse husbandry practices remains high in the modern era. The general rules of stable management have not changed much, while feeding and training systems have undergone modernization but still are based on the historic principles. The Kikkuli training example demonstrates that following the old rules can be beneficial, especially for trainers who seek to improve endurance of horses.

It can be concluded that horse breeding and management are truly the result of the continuous evolution of practices. The importance of the horse is clear from the investment made in their development and enhancement of the scope of their uses. Understanding a history is important to guiding the future development of horse management.

In the context of five millennia of horse domestication, characterized by slow but steady improvements, the intensive selection implemented in the last 200 years, combined with novel reproduction techniques not older than 50 years, raise questions about the future of horse breeding. Breeders are able to achieve the desired phenotypes within a few generations, but this comes with the cost of drastic reduction in genetic diversity. The massive extinction of ancestral breeds in the 20th century is a warning to modern breeders that the dynamic pace of phenotypic improvement can result in irreversible loss in the genetic makeup of the species.

## Figures and Tables

**Table 1:** First noted appearances of selected coat color phenotypes. The genotype column shows the combination of alleles required to express a certain phenotype, with specific alleles written in a superscript.

Phenotype		Locus	Genotype	First Observed
Bay	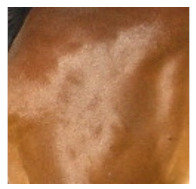	*ASIP*	A^A^/- E^E^/-	Pleistocene [85]
Black	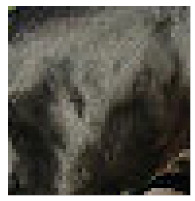	*ASIP*	A^a^/A^a^ E^E^/-	Copper Age [85]
Chestnut	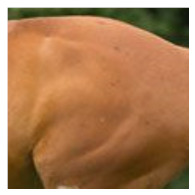	*MC1R*	E^e^/E^e^	4300 BC [87]
Bay leopard	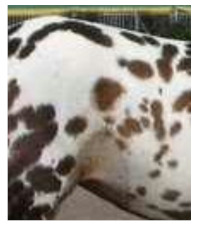	*ASIP* *TRPM1*	A^A^/- E^E^/- LP/-	Pleistocene [83]
Chestnut leopard	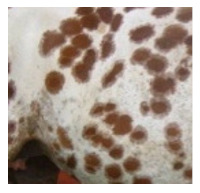	*MC1R* *TRPM1*	E^e^/E^e^ LP/-	2700 years ago [78]

## Data Availability

This study did not report any data.
